# Analysis of the nearly complete mitochondrial genome of *Paederus fuscipes* (Coleoptera: Staphylinidae)

**DOI:** 10.1080/23802359.2017.1422410

**Published:** 2018-01-05

**Authors:** Aili Lin, Nan Song, Xincheng Zhao, Fangmei Zhang

**Affiliations:** a Department of Entomology, College of Plant Protection, Henan Agricultural University, Zhengzhou, People’s Republic of China;; b Department of Entomology, Xinyang Agriculture and Forestry University, Xinyang, People’s Republic of China

**Keywords:** *Paederus fuscipes*, mitochondrial genome, phylogenetic analysis

## Abstract

The nearly complete mitochondrial genome of *Paederus fuscipes* (GenBank accession no. MG581161) is 17,644 bp in size, containing 13 protein-coding genes, 22 transfer RNAs, two ribosomal RNAs, and a partial control region. The gene order is similar to the typical insect mitochondrial genome. Maximum likelihood tree recovered the monophyly of Staphylininae, Pselaphinae, Paederinae and Aleocharinae. Additionally, Staphylininae is a sister group to Paederinae.

The family Staphylinidae, belonging to Coleoptera, contains over 61,000 described extant species distributed worldwide (Newton 2015). We determined the nearly complete mitochondrial genomes of *Paederus fuscipes* to expand the data of mitochondrial genomes of Staphylinidae and to better understand the phylogenetic relationships of Staphylinidae.

*Paederus fuscipes* was collected in Zhengzhou, China (the geospatial coordinates: 113.635°E, 34.723°N), the primary specimen can be obtained by the Entomological Museum of Henan Agricultural University (voucher no. MT-Zz15091901). Total genomic DNA was extracted from muscular tissue preserved in the absolute ethyl alcohol at −20 °C using the TIANamp Micro DNA Kit (Tiangen Biotech Co., Ltd, Beijing, China). We reconstructed the nearly complete mitochondrial genome of *P. fuscipes* from the library composed of the Genomic DNA of *P. fuscipes* and other unrelated insects, by using sequencing of PE 150 in the Illumina HiSeq 2500 platform. The raw reads were de novo assembled by the SOAPdenovo software (Zhao et al. 2011) with an average 358.98 × coverage. We identified the nearly complete mitochondrial genome of *P. fuscipes* from a single large contig of 17,386 bp by blasting the mitochondrial *rrnS* gene bait sequence against the assembled contigs with BioEdit7.0.9.0 (Hall 1999).

The nearly complete mitochondrial genomes of *P. fuscipes* (GenBank accession no. MG581161) is 17,644 bp in length, contains 13 protein-coding genes, 22 transfer RNAs, two ribosomal RNAs, and a partial control region. The gene arrangement is similar to the typical insect mitochondrial genomes (Wolstenholme 1992). All protein-coding genes of *P. fuscipes* start with the start codon ATN except for *nad1*, which begins with putative start codon TTG. Ten protein-coding genes of *P. fuscipes* use TAA or TAG as stop codon, but three of the genes (i.e. *cox2*, *cox3* and *nad4*) use T as incomplete stop codon. All 22 tRNA genes can be folded into the typical cloverleaf structure, but the anticodon of *trnS1* is UCU rather than usual GCU. Although the control region has a length of 3119 bp, it is still incomplete due to the lack of the overlapping sequences to connect two ends of this region.

Phylogenetic analysis shows that Staphylininae, Pselaphinae, Paederinae and Aleocharinae are monophyletic (Brunkea et al. 2015; Mckenna et al. 2015) (Figure 1). Staphylininae is a sister group to Paederinae ( Crowson 1955; Lawrence and Newton 1995; Beutel and Molenda 1997; Assing 2000; Solodovnikov and Newton 2005; Solodovnikov et al. 2013; Mckenna et al. 2015). However, Omaliinae and Tachyporinae are non-monophyletic (Mckenna et al. 2015).

**Figure 1.**
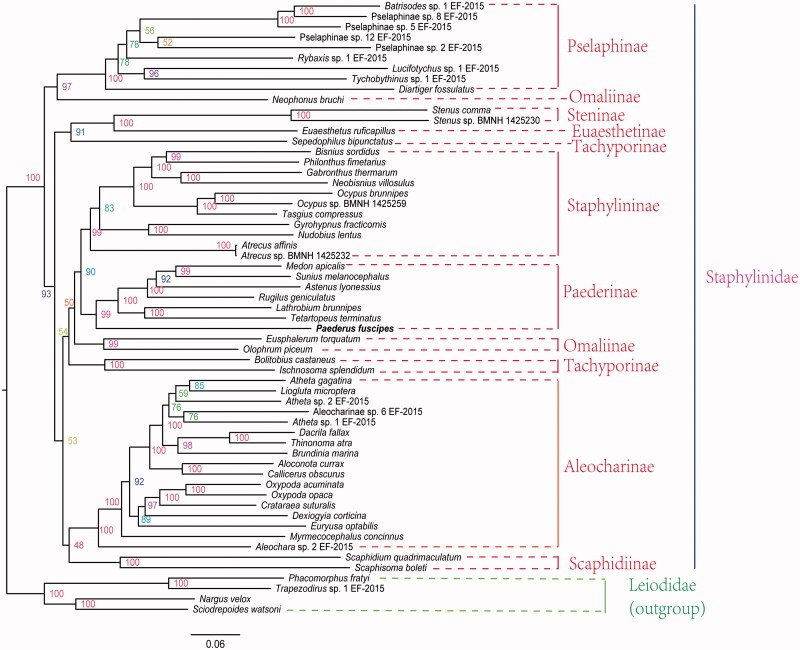
Maximum-likelihood tree of the mitogenomes of *P. fuscipes* and 58 other species from GenBank. The maximum-likelihood analysis was reconstructed by concatenated nucleotide sequences of 13 mitochondrial protein genes (10,869 bp) using IQ-TREE (Nguyen et al. 2015). Numbers alongside nodes refer to bootstrap support values. The name in the rough line is our determined mitochondrial genome. All 59 species accession numbers are listed as below: *Eusphalerum torquatum* (KT780648), *Neophonus bruchi* (KT780663), *Olophrum piceum* (NC_028605), Pselaphinae sp. 5 EF-2015 (KT780684), Pselaphinae sp. 8 EF-2015 (KT780689), Pselaphinae sp. 12 EF-2015 (KT780678), *Batrisodes* sp. 1 EF-2015 (KT780631), *Diartiger fossulatus* (KT780644), *Lucifotychus* sp. 1 EF-2015 (KT780656), *Rybaxis* sp. 1 EF-2015 (KT780672), *Tychobythinus* sp. 1 EF-2015 (KT780701), Pselaphinae sp. 2 EF-2015 (KT780680), *Scaphidium quadrimaculatum* (NC_028609), *Scaphisoma boleti* (KT780674), *Astenus lyonessius* (KT780626), *Lathrobium brunnipes* (KT780654), *P. fuscipes* (MG581161), *Medon apicalis* (KT780658), *Rugilus geniculatus* (NC_028608), *Sunius melanocephalus* (KT780696), *Tetartopeus terminatus* (NC_028613), *Euaesthetus ruficapillus* (KT780646), *Stenus* sp. BMNH 1425230 (KT876913), *Stenus comma* (KT780694), *Atrecus* sp. BMNH 1425232 (KT876882), *Atrecus affinis* (NC_028597), *Neobisnius villosulus* (KT780662), *Philonthus fimetarius* (KT780669), *Bisnius sordidus* (KT780632), *Gabronthus thermarum* (NC_028601), *Ocypus* sp. BMNH 1425259 (KT876908), *Ocypus brunnipes* (KT780665), *Tasgius compressus* (KT780697), *Gyrohypnus fracticornis* (KT780650), *Nudobius lentus* (KT780664), *Atheta gagatina* (KT780629), *Aleochara* sp. 2 EF-2015 (KT780622), Aleocharinae sp. 6 EF-2015 (KT780687), *Aloconota currax* (KT780624), *Atheta* sp. 2 EF-2015 (KT780628), *Atheta* sp. 1 EF-2015 (KT780627), *Callicerus obscurus* (NC_028598), *Liogluta microptera* (NC_028602), *Euryusa optabilis* (NC_028600), *Myrmecocephalus concinnus* (NC_028604), *Dexiogyia corticina* (KT780643), *Oxypoda acuminata* (NC_028606), *Crataraea suturalis* (KT780639), *Oxypoda opaca* (JX412751), *Brundinia marina* (KT780635), *Thinonoma atra* (KT780699), *Dacrila fallax* (NC_028599), *Bolitobius castaneus* (KT780633), *Ischnosoma splendidum* (KT780653), *Sepedophilus bipunctatus* (NC_028611), *Nargus velox* (KT780661), *Trapezodirus* sp. 1 EF-2015 (KT780700), *Phacomorphus fratyi* (NC_028607), *Sciodrepoides watsoni* (NC_028610).
